# Relationship between Activities of Daily Living and Readmission within 90 Days in Hospitalized Elderly Patients with Heart Failure

**DOI:** 10.1155/2017/7420738

**Published:** 2017-10-22

**Authors:** Masahiro Kitamura, Kazuhiro P. Izawa, Hiroki Taniue, Yumi Mimura, Keita Imamura, Hitomi Nagashima, Peter H. Brubaker

**Affiliations:** ^1^Department of Physical Therapy, Kokura Rehabilitation College, 2-2-10 Kuzuharahigashi, Kokuraminami, Kitakyushu 800-0206, Japan; ^2^Graduate School of Health Sciences, Kobe University, 7-10-2 Tomogaoka, Suma, Kobe 654-0142, Japan; ^3^Department of Rehabilitation, Shinyukuhashi Hospital, 1411 Douzyouzi, Yukuhashi 824-0026, Japan; ^4^Department of Health and Exercise Science, Healthy Exercise & Lifestyle Programs, Wake Forest University, 305 Reynolds Gym, Wingate Dr., Winston-Salem, NC 27109, USA

## Abstract

**Aims:**

To examine the relationship between activities of daily living (ADL) and readmission within 90 days and assess the cutoff value of ADL to predict readmission in hospitalized elderly patients with heart failure (HF).

**Methods:**

This cohort study comprised 589 consecutive patients with HF aged ≥65 years, who underwent cardiac rehabilitation from May 2012 to May 2016 and were discharged home. We investigated patients' characteristics, basic attributes, and ADL (motor and cognitive Functional Independence Measure [FIM]). We analyzed the data using the unpaired* t-*test, *χ*^2^ test, Cox proportional hazard model, receiver operating characteristic (ROC) curve, and Kaplan-Meier method.

**Results:**

Of 589 patients, 113 met the criteria, and they were divided into the nonreadmission (*n* = 90) and readmission groups (*n* = 23). Age, body mass index, New York Heart Association class, hemoglobin level, and motor FIM score were significantly different between the two groups (*p* < 0.05). The body mass index (hazard ratio [HR]: 0.87; *p* < 0.05) and motor FIM score (HR: 0.94; *p* < 0.01) remained statistically significant. The cutoff value for the motor FIM score determined by ROC curve analysis was 74.5 points (area under the curve = 0.78; *p* < 0.001).

**Conclusion:**

The motor FIM score in elderly patients with HF was an independent predictor of rehospitalization within 90 days.

## 1. Introduction

Heart failure (HF) affects about 1% of individuals in their 50s and 10% of those in their 80s, and its incidence is increasing rapidly with age worldwide [1]. Among individuals with HF, problems such as an increase in the readmission rate and medical expenses and many readmissions in the short-term occur [2, 3]. In epidemiological studies conducted among the Japanese elderly patients with HF, complications in general, increase in the length of hospital stay, high readmission rates, and increased medical expenses have been reported [4]. Among patients who are readmitted, there are increased cases of disease onset other than heart disease [5]. The readmission rate is high within 6 months [3, 6]. Age, severity, length of hospital stay, comorbidities, and disease management are the risk factors for short-term readmission [7–10].

One of the purposes of rehabilitation in patients with HF is the recovery of activities of daily living (ADL) [11]. ADL and functional limitations in patients with HF are associated with readmission [12, 13]. Few studies have shown the relationship between readmission and ADL in patients with HF, and the cutoff values of ADL to predict readmission are unknown.

Therefore, we assumed that, in patients with HF, the group with poor ADL would have a higher readmission rate than the group with good ADL. The purpose of the present study was to investigate the relationship between ADL and readmission within 90 days in elderly patients with HF.

## 2. Materials and Methods

### 2.1. Study Design and Participants

Five hundred eight-nine consecutive patients with HF who underwent rehabilitation at one acute care hospital from May 2012 to May 2016 were included in this retrospective cohort study. Of these patients, those aged ≥65 years and those who could walk with assistance before hospitalization and during the initial hospitalization were included. Patients who underwent pacemaker operation during hospitalization, those who were transferred to other departments, those who were not discharged home, those who died during hospitalization, and those who were difficult to follow for 90 days were excluded from this study. The reason for excluding patients with a pacemaker was because the rehabilitation protocol for these patients is different.

The Kokura Rehabilitation College Institutional Review Committee on Human Research approved this study (approval number 29-03), and informed consent was obtained from each patient.

### 2.2. Rehabilitation after Hospitalization

Patients in this study received rehabilitation in accordance with the Japanese guidelines [11]. After confirming with the doctor that the patient experienced no symptoms with light activity, we encouraged rehabilitation, such as the sitting position, standing position, walking, and ADL. If patients were able to walk, we recommended aerobic exercise to increase endurance necessary for them to perform ADL.

### 2.3. Investigation

Patients' characteristics and clinical parameters, including age, sex, body mass index (BMI), left ventricular ejection fraction (LVEF), brain natriuretic peptide (BNP) concentration, New York Heart Association (NYHA) class at discharge, estimated glomerular filtration rate (eGFR), creatinine level at discharge, hemoglobin level at discharge, albumin level at discharge, acute management, comorbidity, Charlson comorbidity index, medications, time of initiation of walking exercise, length of hospital stay, motor Functional Independence Measure (FIM) score at discharge, and cognitive FIM score at discharge, were evaluated by reviewing medical records retrospectively. We divided patients into two groups, the nonreadmission or readmission group within 90 days, based on a previous study [9, 10]. We also evaluated the FIM as a measurement of ADL [14].

### 2.4. Assessment of ADL

The FIM was developed to suit rehabilitative aspects of patients with disabilities, and it consisted of two domains: motor and cognitive [14]. The motor domain (motor FIM) consists of 13 items: eating; grooming; bathing; dressing upper body; dressing lower body; toileting; bladder management; bowel management; transfer to bed, chair, or wheelchair; transfer to toilet; transfer to tub or shower; walking/wheelchair; and stairs. The cognitive domain (cognitive FIM) consists of 5 items: comprehension, expression, social interaction, problem solving, and memory. A scoring scale from 1 to 7 points was used (1 point for total assistance, 2 points for maximal assistance, 3 points for moderate assistance, 4 points for minimal contact assistance, 5 points for supervision, 6 points for modified independence, and 7 points for complete independence). The minimum total FIM score was 18 points, and the maximum total FIM score was 126 points, whereas the minimum scores for the motor FIM and cognitive FIM were 13 points and 5 points, and maximum scores for the motor FIM and cognitive FIM were 91 points and 35 points, respectively. This measurement was obtained by two physical therapists from the time of discharge.

### 2.5. Assessment of Follow-Up

Patients enrolled in this study were followed up for 90 days. The first follow-up clinic visit was scheduled within the first 2 weeks after discharge. The following readmission information was obtained from medical records by two physical therapists: the date of readmission, number of days from discharge to readmission, and reasons for readmission. The definition of readmission was admission for all causes within 90 days after discharge, except hospitalization for examination.

### 2.6. Statistical Analysis

Patients' characteristics and clinical parameters were reported using percentages for categorical variables and the mean ± standard deviation for continuous variables. The unpaired* t*-test and chi-square test were used to compare patients' characteristics and clinical parameters between the two groups. A Cox proportional hazard model for readmission within 90 days was used to ascertain whether ADL at discharge was an independent predictor of readmission within 90 days (hazard ratio and 95% confidence interval). The objective variables used in this model were readmission (the end point), data 0 (nonreadmission), and data 1 (readmission). The explanatory variables used in this model were variables that showed statistical significance at *p* < 0.05 in univariate analysis. The detailed items between the two groups that were significant in these analyzes were examined. To determine the cutoff value of the most influential factor obtained by these analyses, a receiver operating characteristic (ROC) curve was constructed by plotting the sensitivity against the false positive rate. Patients were classified into two groups according to these cutoff values, a Kaplan-Meier curve was constructed, and a log-rank test was used. A* p* value < 0.05 indicated statistical significance. Statistical analyses were performed using IBM SPSS 23.0 J statistical software (IBM SPSS Japan, Inc., Tokyo, Japan).

## 3. Results

### 3.1. Flow of Included Patients

**Figure 1:**
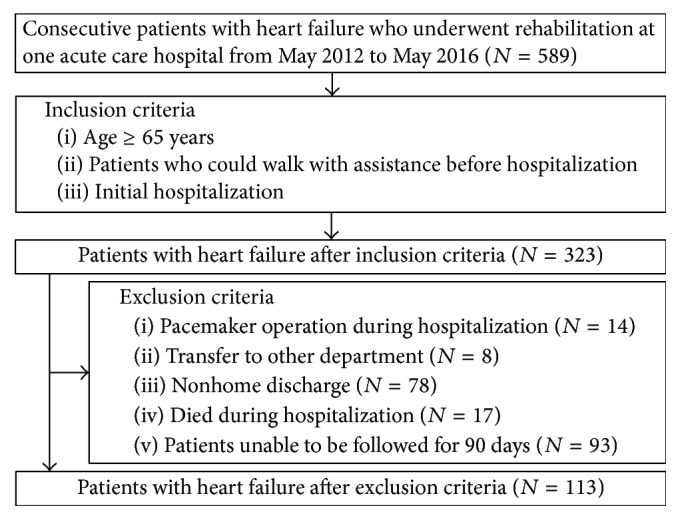


A flow chart of patients included in this study is shown in Figure 1. Of 589 consecutive patients with HF who underwent rehabilitation, 323 met the inclusion criteria, but 210 patients were excluded later because of pacemaker operation during hospitalization (14), transfer to other departments (8), nonhome discharge (78), death during hospitalization (17), or difficulty to follow up for 90 days (93 patients). Therefore, 113 patients were ultimately included and divided into the nonreadmission group (*n* = 90) or readmission group (*n* = 23).

### 3.2. Patients' Characteristics

A comparison of the patients' clinical characteristics between the nonreadmission group and readmission group is shown in Table 1. Compared to the nonreadmission group, the readmission group was significantly older and had a lower BMI, poorer NYHA class, lower hemoglobin level at discharge, and lower motor FIM score (*p* < 0.05).

### 3.3. Factor of Readmission

Results of the Cox proportional hazard models, as provided in Table 2, demonstrate the associations between each parameter and readmission within 90 days. In the univariate Cox proportional hazard model with age, variables like BMI, NYHA class at discharge, hemoglobin level at discharge, and motor FIM score at discharge as covariates were independent predictors of readmission. In the multivariate Cox proportional hazard model with age, BMI, NYHA class at discharge, hemoglobin level at discharge, and motor FIM score at discharge as covariates, BMI (hazard ratio: 0.87; 95% confidence interval: 0.76–0.99), and motor FIM score at discharge (hazard ratio: 0.94; 95% confidence interval: 0.89–0.99) were independent predictors of readmission (Table 3). A comparison of motor FIM items between the groups is shown in Table 4.

### 3.4. Cutoff Value of the Motor FIM Score for Predicting Readmission

**Figure 2:**
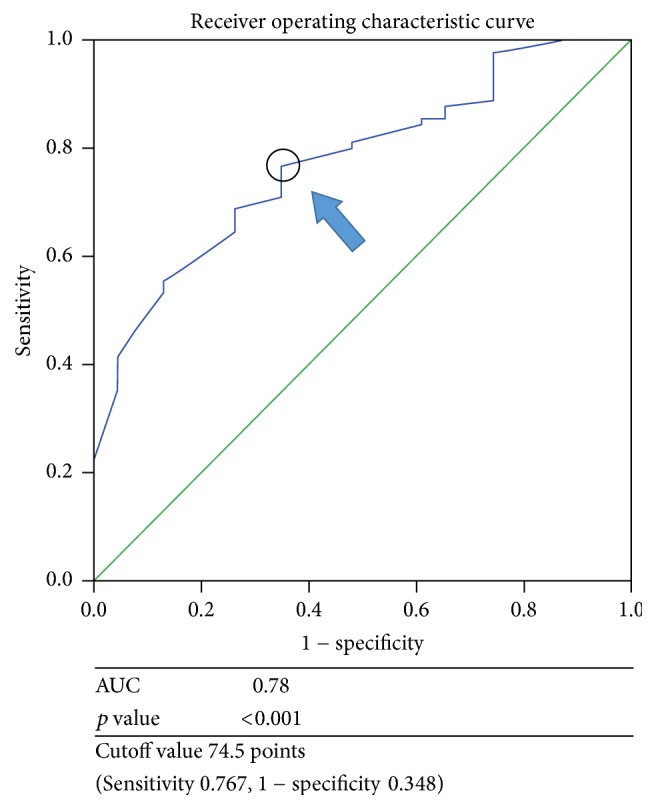


The cutoff value of the motor FIM score at discharge that predicted the occurrence of readmission in the ROC curve was 75 points (area under the curve: 0.78, *p* < 0.001, sensitivity: 0.767, false positive rate: 0.348) (Figure 2).

### 3.5. Readmission Rates Based on the Motor FIM Score

**Figure 3:**
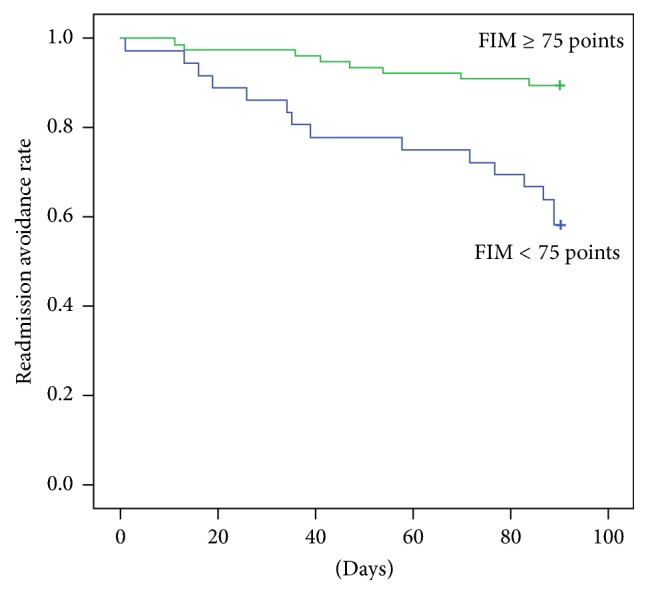


In the Kaplan-Meier analysis, we divided patients into two groups based on the cutoff values of the motor FIM score. The group with a motor FIM score ≥ 75 points had significantly higher readmission avoidance rates than the group with a motor FIM score < 75 points (log-rank test, *p* < 0.001) (Figure 3).

## 4. Discussion

To our knowledge, this is the first study to report the differences in motor ADL in elderly hospitalized patients with HF that are associated with readmission within 90 days.

### 4.1. Characteristics of the Readmission Group of Elderly Patients with HF

The elderly patients with HF in the readmission group were significantly older with a poorer NYHA class, lower hemoglobin level, and lower motor FIM score than those in the nonreadmission group. These findings were largely in agreement with the characteristics of patients with HF who were readmitted in previous studies. In a past study, an older age and low BMI in patients with HF were risk factors for short-term readmission [15]. In patients with HF, a low BMI is known to reduce readmission [16]. Additionally, the poor NYHA class is associated with readmission within 90 days [17]. Anemia in patients with HF is a predictor of readmission within 90 days [9, 18], and their low hemoglobin levels are likely to result in readmission because of heart load [19, 20]. Additionally, the low ADL in patients with HF is associated with readmission within 30 days [21]. However, the readmission rate (20.4%) within 90 days in this study is lower than that reported in these aforementioned previous studies. The subjects in this study included those who could walk and were hospitalized for the first time; those with a nonhome discharge were excluded. Based on these criteria, there were many patients with HF with a good condition, which is why the readmission rate may be low. Therefore, in our study, although subjects' readmission rate was low, the characteristics of patients with HF are almost consistent with those of previous studies; thus, these patient characteristics are considered partially generalizable.

### 4.2. Relationship between Readmission and Motor ADL

In patients with HF who were readmitted because of poor ADL recovery during hospitalization and declining ADL after discharge, new events may occur due to an increased heart load. Low ADL at discharge in patients with internal disorders is associated with a high readmission rate, and change in the rate of ADL during hospitalization is related to readmission. Intervention to prevent ADL decline during hospitalization is important [22]. Patients with HF after discharge are likely to show a decline in physical function, with the possibility of readmission due to events such as disease and falls [23, 24]. In addition, in patients with a disability who underwent rehabilitation during hospitalization, a low ADL at discharge was associated with a high rate of readmission within 90 days, and HF was a risk factor of complications [25]. Therefore, in patients with hospitalization, physicians need to conduct further research on ADL during hospitalization and the status of ADL after discharge. In the readmission group, low motor FIM scores were for self-care, transfer, and locomotion. Walking is known as a readmission factor in patients with HF [26]. Self-care is reported as a prognostic factor in elderly hospitalized patients [27]. Improvements in items such as self-care, transfer, and locomotion shown in this study may prevent readmission. It is important to investigate the relationship between physical function and ADL to prevent readmission in the future.

### 4.3. Clinical Implication

Motor ADL was an independent factor of readmission within 90 days in elderly patients with HF. Improvement of ADL at discharge may reduce readmission. The cutoff value of the motor FIM score may be an indicator for readmission. These findings suggest the importance of intervention to improve ADL during hospitalization and after discharge.

### 4.4. Limitations

This retrospective cohort study was conducted at one facility with a small sample. Based on the inclusion and exclusion criteria, only approximately 20% of the hospitalized patients with HF were study subjects. The motor FIM may also have a ceiling effect [28]. Moreover, we did not consider the differences according to sex in this study. Additionally, we did not investigate the difference in physical function according to sex [12, 29], and we were unable to follow up with some patients and to examine the clinical characteristics and ADL between patients who were and were not discharged home. Further, we did not assess outpatient rehabilitation after discharge.

## 5. Conclusion

The motor ADL score in elderly patients with HF was an independent factor of readmission, and its cutoff value was 74.5 points.

## Figures and Tables

**Table 1:** Patients' characteristics.

	Nonreadmission *n* = 90	Readmission *n* = 23	*F* or *χ*^2^ value	*p* value
Age, years	79.6 ± 6.9	83.8 ± 5.9	1.12^a^	0.008
Sex, male, %	55.6	47.8	0.44	0.51
BMI, kg/m^2^	22.8 ± 3.0	21.3 ± 3.5	0.89^a^	0.04
Clinical parameter				
LVEF, %	47.2 ± 16.6	49.4 ± 13.5	2.13^a^	0.55
BNP level, pg/mL	783.8 ± 826.4	696.2 ± 410.2	2.83^a^	0.62
NYHA class I/II, %	84.4/15.6	65.2/34.8	4.32	0.04
Creatinine level, mg/dL	1.3 ± 0.9	1.9 ± 1.7	9.43	0.16
eGFR, mL/min/1.73 m^2^	50.1 ± 21.8	40.3 ± 24.7	0.92^a^	0.06
Hemoglobin level, g/dL	11.5 ± 2.0	10.4 ± 2.2	0.13^a^	0.04
Albumin level at discharge, g/dL	3.5 ± 0.6	3.4 ± 0.4	0.14	0.35
Acute management, %	18.9	8.7	1.36	0.24
Comorbidity, %				
Hypertension	86.7	82.6	0.25	0.62
Diabetes	44.4	30.4	1.48	0.22
Ischemic heart disease	51.1	47.8	0.08	0.78
Valvular disease	27.8	43.5	2.11	0.15
Atrial fibrillation	41.1	56.5	1.76	0.18
Orthopedic disease	37.8	34.8	0.07	0.79
Neurological disease	24.0	5.6	3.04	0.08
Respiratory disease	21.1	21.7	0.004	0.95
CCI	2.2 ± 1.9	2.8 ± 2.3	1.82	0.45
Medication				
Diuretic	93.3	95.7	0.17	0.68
*β*-blockers	58.9	52.2	0.34	0.56
ACEI/ARB	40.0	47.8	0.46	0.50
Rehabilitation progress				
Initiation of walking exercise, days	4.6 ± 4.9	6.2 ± 7.1	3.15^a^	0.21
Length of hospital stay, days	17.3 ± 7.4	15.9 ± 7.1	0.23^a^	0.43
Motor FIM score on admission	39.4 ± 18.0	34.8 ± 15.2	0.85^a^	0.26
Motor FIM score at discharge	79.8 ± 8.1	70.9 ± 9.5	0.94^a^	<0.001
Cognitive FIM score on admission	29.6 ± 7.0	26.5 ± 8.2	0.61^a^	0.07
Cognitive FIM score at discharge	33.0 ± 3.9	31.2 ± 5.2	4.78^a^	0.08

Values are presented as a mean ± standard deviation or %; ACEI = angiotensin-converting enzyme inhibitor; ARB = angiotensin receptor blocker; BMI = body mass index; BNP = brain natriuretic peptide; CCI = Charlson Comorbidity Index; eGFR = estimated glomerular filtration rate; FIM = Functional Independence Measurement; LVEF = left ventricular ejection fraction; NYHA = New York Heart Association. ^a^*F* value.

**Table 2:** Results of univariate analysis.

	Cox proportional hazard ratio	95% CI		*p* value
Age, years	1.09	1.02	1.17	0.01
BMI, kg/m^2^	0.86	0.75	0.98	0.03
NYHA class at discharge I/II, %	2.53	1.07	5.96	0.03
Hemoglobin level at discharge, g/dL	0.76	0.61	0.95	0.02
m-FIM score at discharge	0.92	0.89	0.96	<0.001

BMI = body mass index; CI = confidence interval; m-FIM = motor Functional Independence Measurement; NYHA = New York Heart Association.

**Table 3:** Results of multivariate analysis.

	Cox proportional hazard ratio	95% CI		*p* value
Age, years	1.02	0.94	1.10	0.70
BMI, kg/m^2^	0.87	0.76	0.99	0.047
NYHA class at discharge I/II, %	1.52	0.61	3.77	0.28
Hemoglobin level at discharge, g/dL	0.88	0.70	1.11	0.37
m-FIM score at discharge	0.94	0.89	0.99	0.012

BMI = body mass index; CI = confidence interval; m-FIM = motor Functional Independence Measurement; NYHA = New York Heart Association.

**Table 4:** Comparison of FIM items between the nonreadmission and readmission groups.

	Nonreadmission group *n* = 90	Readmission group *n* = 23	*F* or *χ*^2^ value	*p* value
Eating	6.8 ± 0.6	6.7 ± 0.5	0.46^a^	0.430
Grooming	6.6 ± 0.8	6.0 ± 1.0	1.39^a^	0.004
Bathing	6.2 ± 1.1	5.2 ± 1.2	0.04^a^	0.001
Dressing upper body	6.5 ± 0.8	5.7 ± 1.0	0.36^a^	<0.001
Dressing lower body	6.5 ± 0.9	5.6 ± 1.1	1.14^a^	<0.001
Toileting	6.5 ± 0.8	5.8 ± 1.2	3.42^a^	0.002
Bladder management	6.8 ± 0.6	6.5 ± 0.8	8.74^a^	0.100
Bowel management	6.8 ± 0.6	6.4 ± 1.1	21.09^a^	0.060
Transfer to bed/chair/wheelchair	6.7 ± 0.6	6.4 ± 0.7	1.96^a^	0.044
Transfer to toilet	6.6 ± 0.7	6.1 ± 0.9	0.54^a^	0.005
Transfer to tub/shower	5.2 ± 1.7	4.2 ± 1.5	1.10^a^	0.012
Walking/wheelchair	6.3 ± 0.9	5.6 ± 1.3	0.60^a^	0.007
Stairs	2.4 ± 2.1	1.4 ± 1.2	17.55^a^	0.005

Values are presented as a mean ± standard deviation; ^a^*F* value.

## Abbreviations

ACEI: Angiotensin-converting enzyme inhibitor
ADL: Activities of daily living
ARB: Angiotensin receptor blocker
BMI: Body mass index
BNP: Brain natriuretic peptide
eGFR: Estimated glomerular filtration rate
FIM: Functional Independence Measure
HF: Heart failure
LVEF: Left ventricular ejection fraction
NYHA: New York Heart Association.
